# Inducible orthogonal aminoacylation demonstrates that charging is required for mitochondrial tRNA import in *Trypanosoma brucei*

**DOI:** 10.1038/s41598-019-47268-4

**Published:** 2019-07-25

**Authors:** Jonathan L. Huot, Shikha Shikha, André Schneider

**Affiliations:** Department of Chemistry and Biochemistry, University of Bern, Freiestrasse 3, Bern, CH-3012 Switzerland

**Keywords:** RNA transport, Molecular biology, RNA metabolism

## Abstract

Orthogonal aminoacyl-tRNA synthetase/tRNA pairs have emerged as powerful means of site-specifically introducing non-standard amino acids into proteins *in vivo*. Using amino acids with crosslinking moieties this method allows the identification of transient protein-protein interactions. Here we have introduced a previously characterized evolved tyrosyl-tRNA synthetase/suppressor tRNA^Tyr^ pair from *E. coli* into the parasitic protozoan *Trypanosoma brucei*. Upon addition of a suitable non-standard amino acid the suppressor tRNA^Tyr^ was charged and allowed translation of a green fluorescent protein whose gene contained a nonsense mutation. - *T. brucei* is unusual in that its mitochondrion lacks tRNA genes indicating that all its organellar tRNAs are imported from the cytosol. Expression of the bacterial tyrosyl-tRNA synthetase in our system is tetracycline-inducible. We have therefore used it to demonstrate that cytosolic aminoacylation of the suppressor tRNA^Tyr^ induces its import into the mitochondrion.

## Introduction

Using model organisms to study general biological processes is a cornerstone of cell and molecular biology. However most studies in eukaryotes focus on a rather small number of systems such as yeast, mammals and other animals. This is problematic since it has become clear that eukaryotes are divided into a small number of highly diverged supergroups. With the exception of plants, the common model systems all belong to the same eukaryotic supergroup of the opisthokonts^1,2^. Thus for many cell biological processes in eukaryotes it is not known how conserved they are. One problem is that most non-opisthokonts are not amenable to biochemical and molecular genetic methods. However *Trypanosoma brucei*, a member of the excavate supergroup that is essentially unrelated to plants and opisthokonts, represents an exception. It can be grown in quantities amenable to biochemistry and a whole range of molecular genetic methods including gene replacements by homologous recombination, inducible gene expression^3^ and RNAi^4^ have been established for the system. Moreover, high quality genome, transcriptome and proteome data are available^5^. Thus besides its clinical importance as the causative agent of human sleeping sickness and animal diseases, *T. brucei* is one of very few well established non-opisthokont model systems^6,7^. Indeed a survey of the literature shows that research with trypanosomes has been very rewarding. It led to the discovery of glycosyl-phosphatidyl-inositol-mediated membrane anchoring of proteins, trans-splicing and RNA editing well before these processes were shown to occur in all eukaryotes^8^. On the other hand it was shown that structures thought to be highly conserved in all eukaryotes such as the kinetochore^9^, the mitochondrial protein import machinery^10^ and the mitochondrial ribosome^11^ look very different in trypanosomes than in other eukaryotes.

Since many years trypanosomes have also been used as a model to study tRNA biology such as tRNA modifications and mitochondrial tRNA import^12–14^. The main reason for the latter is that the mitochondrial genome of trypanosomes, unlike in most other eukaryotes, does not encode any tRNA genes. As a consequence all mitochondrial tRNAs derive from cytosolic tRNAs, a small fraction of which is imported into the mitochondrion. Recently it has been shown that the core subunits of the mitochondrial outer membrane protein import machinery also mediate tRNA import. The two protein import receptors however are not required for the process, indicating that while tRNAs and proteins use the same pore for translocation across the outer membrane the two processes are not coupled to each other^15^.

In the present study we have expanded the repertoire of molecular genetic methods for *T. brucei*. We have devised a system allowing site-specific insertion of non-biological amino acids in a protein of interest. The system is based on an orthogonal tRNA/aminoacyl-tRNA synthetase (aaRS) pair and works as outlined below.

**Figure 1:**
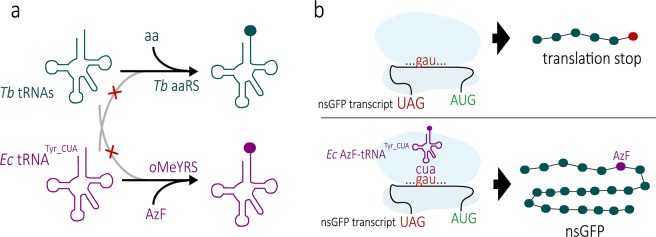
Orthogonal aaRS and tRNA pair allows site-specific insertion of non-natural amino acids into a target protein. (**a**) The *E. coli* oMeYRS/tRNA^Tyr_CUA^ pair is orthogonal in eukaryotes including trypanosomes (*Tb*) as the pair is not recognized by standard eukaryotic aaRSs and tRNAs, respectively. Moreover, it was evolved to specifically use non-natural tyrosine analogues (e. g. AzF) for charging. (**b**) Expression of a GFP variant whose gene is interrupted by a stop codon (nsGFP) was used to test whether AzF can be inserted into a protein at a site determined by an internal UAG stop codon.

The genetic code specifies the twenty canonical amino acids in varying degrees of redundancy using 61 codons. The three remaining codons signal for a translation stop, and where one of these appears within an open reading frame, premature termination of translation occurs. Suppression of the stop codon can be exploited to allow site-specific insertion of a non-natural amino acid into proteins. To that end a mutant aaRS which specifically uses non-natural amino acids and the corresponding suppressor tRNA that recognizes the stop codon are expressed in the system of choice. Formation of an aminoacylated suppressor tRNA then allows insertion of the non-natural amino acid at the position where the stop codon has been introduced into the gene of interest (Fig. 1). For the system to work the aaRS and tRNA used must be as orthogonal as possible to the organism into which they are inserted^16–18^. This means: (i) the introduced aaRS should be specific for both the introduced suppressor tRNA and the non-natural amino acids and (ii) none of the endogenous aaRSs should recognize the suppressor tRNA.

Here we have expressed a previously described evolved bacterial tyrosyl-tRNA synthetase (YRS)/suppressor tRNA^Tyr_CUA^ pair in *T. brucei*^19^. We show that the system results in orthogonal aminoacylation of the introduced tRNA with non-natural amino acids, one of which contains a photocrosslinkable moiety. Moreover it is demonstrated that orthogonal aminoacylation of the tRNA^Tyr_CUA^ allows insertion of the non-natural amino acids at defined sites of an enhanced green fluorescent protein containing a nonsense mutation. Subsequently, we have used the system to investigate the importance of aminoacylation for *in vivo* import of tRNAs into mitochondria.

## Results

### Expression of the orthogonal *E. coli* oMeYRS and tRNA^Tyr_CUA^ in *T. brucei*

The *E. coli* YRS and tyrosine suppressor tRNA^Tyr_CUA^ pair was the first aaRS/tRNA pair identified as being orthogonal in eukaryotic cells^20,21^. Subsequent directed evolution was used to obtain a YRS with decreased specificity for tyrosine, but increased specificity for a variety of non-natural amino acids^22^. Here we have introduced the well-described evolved *E. coli* o-methyltyrosyl-tRNA synthetase (oMeYRS)/tRNA^Tyr_CUA^ pair into *Trypanosoma brucei*^19^ (Fig. 1a). In order to test whether the system works as intended we furthermore transfected the cells with a construct encoding an enhanced green fluorescent protein gene containing a UAG stop codon in the middle of its open reading frame, termed nsGFP. Using this setup, expression of full length nsGFP should only be possible if the growth medium contains L-azidophenylalanine (AzF) or o-methyltyrosine, both of which are substrates of oMeYRS. (Fig. 1b).

**Figure 2:**
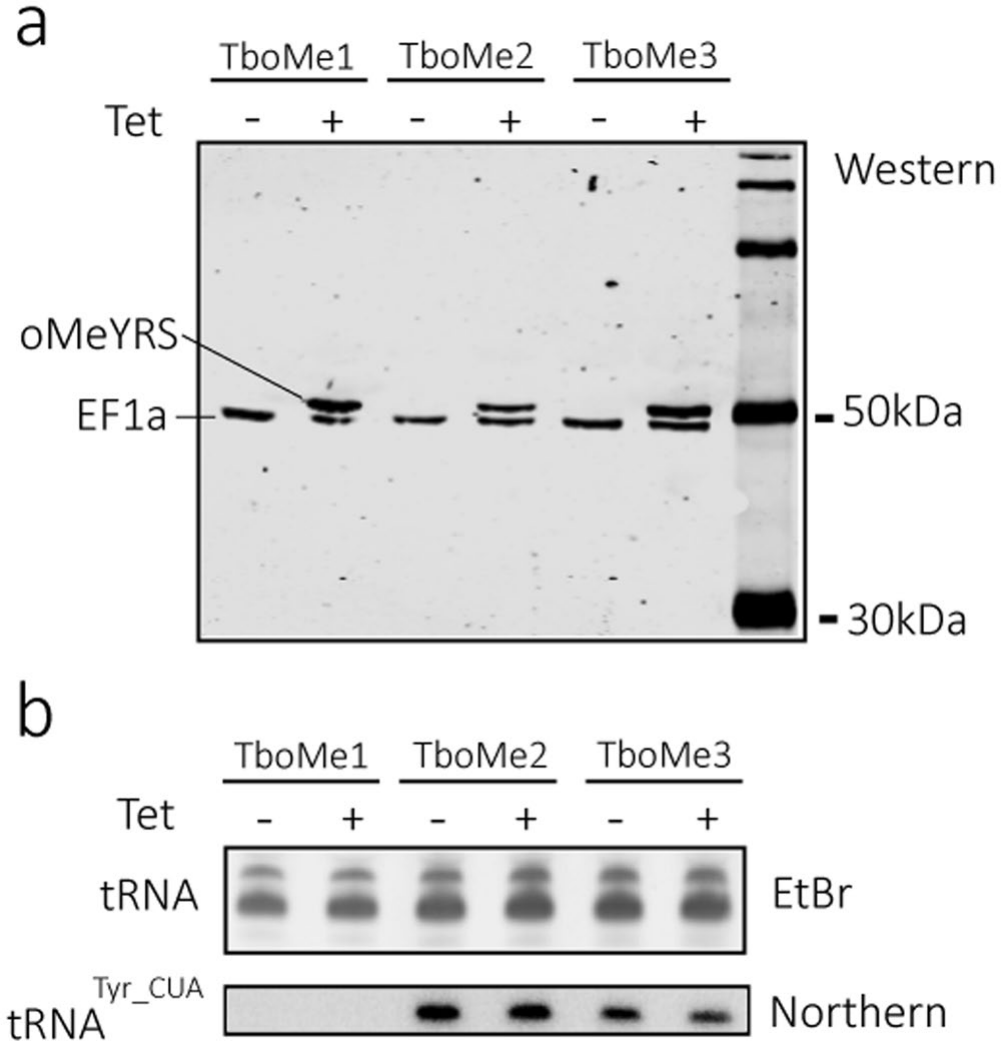
The orthogonal *E. coli* oMeYRS/tRNA^Tyr_CUA^ pair can be expressed in *T. brucei*. (**a**) Immunoblot of total cellular extracts of the indicated cell lines was probed for the cMyc-tagged oMeYRS and EF1a, which served as a loading control. (**b**) Top panel, total RNA extracts from the same cells as in (**a**) were separated on a polyacrylamide gel containing 8 M urea and stained with ethidium bromide. Only the region containing the tRNAs is shown. Bottom panel, Northern blot of the same gel shown in the top panel probed for the tRNA^Tyr_CUA^.

In a first step procyclic *T. brucei* 29-13^3^, which constitutively expresses the tetracycline repressor, was transfected with a construct allowing inducible expression of a cMyc-tagged version of the mutant *E. coli* oMeYRS gene, resulting in the cell line termed TboMe1. The immunoblot in Fig. 2a shows that the oMeYRS was expressed provided that tetracycline was added to the culture. Cytosolic elongation factor 1a (EF1a) serves as a loading control. Subsequently, the TboMe1 cell line was transfected with the gene encoding the *E. coli* suppressor tRNA^Tyr_CUA^ creating the cell line TboMe2. The Western and Northern blots in Fig. 2a,b show that both the oMeYRS and the tRNA^Tyr_CUA^ are expressed in the TboMe2 cell line. The ethidium bromide-stained tRNA portion of the gel serves as a loading control for the Northern analysis. Interestingly, expression of tRNA^Tyr_CUA^ was constitutive, despite the presence of an upstream procyclin promoter that is followed by two tetracycline operators^3^. Thus, it is likely that the bacterial tRNA^Tyr_CUA^ gene contains cryptic intragenic RNA Polymerase III promoter elements. Finally, the TboMe2 cell line was transfected again to introduce the gene for nsGFP containing the in frame UAG stop codon, resulting in the cell line TboMe3. As in TboMe2 both the OMeYRS and tRNA^Tyr_CUA^ are expressed in this cell line (Fig. 2).

### Orthogonal aminoacylation of tRNA^Tyr_CUA^ in *T. brucei*

**Figure 3:**
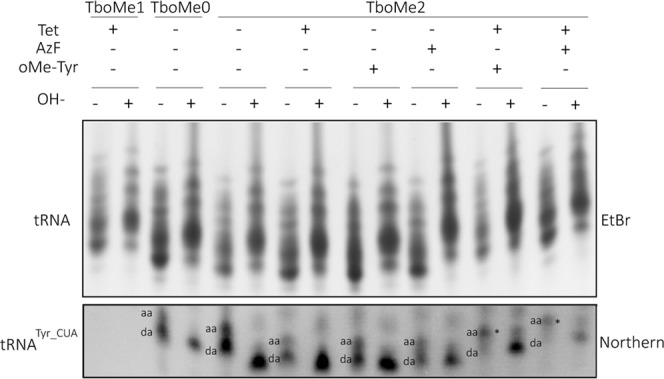
Expression of oMeYRS and addition of o-methyltyrosine or AzF allow aminoacylation of *E. coli* tRNA^Tyr_CUA^ in *T. brucei*. Acylated and deacylated (−/+OH-) total RNA from the indicated uninduced and induced (−/+Tet) cells lines (TboMe1, TboMe0 and TboMe2) grown with or without AzF or o-methyltyrosine (oMe-Tyr) was separated on a long acidic polyacrylamide gel. Top panel, ethidium bromide-stained gel. Only the region containing the tRNAs is shown. Bottom panel, corresponding Northern blot probed for tRNA^Tyr_CUA^. The positions of acylated (aa) and deacylated (da) tRNAs are indicated. The tRNA^Tyr_CUA^ charged by AzF or o-methyltyrosine, respectively, is indicated by asterisks.

In order to test whether the mutant *E. coli* suppressor tRNA^Tyr_CUA^ expressed in *T. brucei* can be aminoacylated we set up cultures of the TboMe1, TboMe2 and TboMe3 cell lines as well as of an additional cell line, that only expresses the tRNA^Tyr_CUA^ (termed TboMe0) but neither oMeYRS nor nsGFP. The cultures were grown in the presence or absence of tetracycline and the non-natural amino acids AzF or o-methyltyrosine as indicated (Figs 3, S2). Subsequently total RNA was extracted and separated on an acid urea polyacrylamide gel which allows separation of aminoacylated tRNA from their faster migrating uncharged forms (Fig. 3)^23^. The ethidium bromide-stained tRNA region of the gel served as a loading control. After transfer of the gel the corresponding blot was probed by oligonucleotide hybridization for the presence of the tRNA^Tyr_CUA^. In order to have a marker for deacylated tRNA^Tyr_CUA^ half of each sample was chemically deacylated by incubation under alkaline conditions (+OH^−^) and separated along with the untreated sample (−OH^−^).

The results show that a higher percentage of aminoacylated tRNA^Tyr_CUA^ was observed in presence of tetracycline when oMeYRS was expressed and when simultaneously AzF or o-methyltyrosine was added to the cell culture (Fig. 3, asterisks). However, significant endogenous aminoacylation of tRNA^Tyr_CUA^ was also detected in all conditions where oMeYRS was absent, and/or when no exogenously added non-natural amino acid was present. This is consistent with the endogenous aminoacylation previously reported when the tRNA^Tyr_CUA^ is expressed in *S. cerevisiae* or *C. albicans*^24^. - In summary these results show that expressing oMeYRS and the tRNA^Tyr_CUA^ in *T. brucei* allows at least partial orthogonal aminoacylation of the *E. coli* suppressor tRNA^Tyr_CUA^ provided that a suitable non-natural amino acid is added to culture medium.

### AzF can be inserted into nsGFP

**Figure 4:**
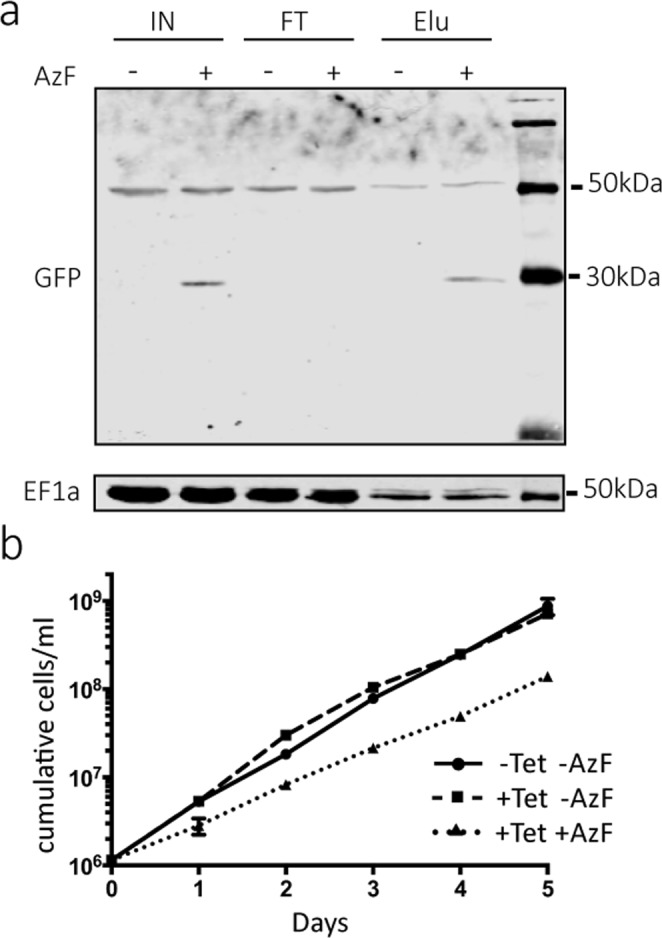
oMeYRS and *E. coli* tRNA^Tyr_CUA^ allow AzF-dependent expression of the nsGFP that is interrupted by a stop codon. **(a)** Total cellular extracts (IN) from the tetracycline-induced TboMe3 cell line (expressing oMeYRS, tRNA^Tyr_CUA^ and the nsGFP mRNA) grown in the absence and presence of AzF, was subjected to metal affinity purification using IMAC resin resulting in a flow through (FT) and eluate fraction (Elu). All fractions were analyzed by immunoblots. Top panel, nsGFP was detected using anti-GFP antibodies. The bands in the top panel at around 50 kDa are a non-specific signal corresponding to the overexpressed oMeYRS that stem from the secondary antibody. Bottom panel, the immunoblot shown in the upper panel was reprobed with a monoclonal EF1a antibody. **(b)** Growth of the TboMe2 cell line was monitored with or without tetracycline (−/+Tet) in the presence or absence of AzF (−/+AzF) as indicated.

The TboMe3 cell line constitutively expresses the *E. coli* suppressor tRNA^Tyr_CUA^, while addition of tetracycline allows expression of oMeYRS, and transcription of the nsGFP mRNA containing a UAG stop codon in the middle of its coding sequence. Figure 4a (IN) shows that further adding AzF to TboMe3 allowed translational readthrough of the nonsense mutation-containing nsGFP, which was C-terminally His-tagged. Moreover the tagged protein could be enriched by immobilized metal affinity chromatography, demonstrating that the readthrough product included the C-terminal tag of nsGFP. No suppression was observed in the absence of added AzF, despite the limited endogenous aminoacylation previously observed (Fig. 3).

The readthrough that is observed in cell lines expressing oMeYRS and tRNA^Tyr_CUA^ in the presence of AzF is not specific for nsGFP but will affect all UAG stop codons. This in turn might affect fitness of the cells. Indeed we observe a reduction of the growth rate in the TboMe2 cell line, that expresses oMeYRS and tRNA^Tyr_CUA^, provided that AzF is added to the culture (Fig. 4b). In the absence of AzF no significant growth difference is observed irrespective of whether the cells express oMeYRS or not, suggesting that efficient readthrough only occurs in the presence of AzF (the same results were obtained for the TboMe3 cell line).

Taken together with the demonstrated inducible aminoacylation of tRNA^Tyr_CUA^ with AzF (Fig. 3) these results show that the *E. coli* suppressor tRNA^Tyr_CUA^ expressed in *T. brucei* can be transcribed and properly processed to allow its function in translation elongation.

### Aminoacylation with AzF activates mitochondrial import of tRNA^Tyr_CUA^

Tetracycline-inducible aminoacylation of the *E. coli* suppressor tRNA^Tyr_CUA^ by AzF offers a way to investigate the role aminoacylation plays in mitochondrial tRNA import. Except for the cytosol-specific tRNA^Met-i ^^25^ and tRNA Sec-JLH ^26^ a variable fraction of all trypanosomal tRNAs are imported into mitochondria. We therefore wanted to see whether and if under which conditions the tRNA^Tyr_CUA^ can be imported into the mitochondrion. To that end the cell lines TboMe2 and TboMe3 were induced by tetracycline in the presence and absence of AzF. Subsequently RNA was extracted from cytosolic and crude mitochondrial fractions, separated on a denaturing polyacrylamide gel and stained by ethidium-bromide. The exclusive presence of cytosolic and mitochondrial rRNAs in their respective fractions illustrates the quality of the cell fractionation. Subsequently the blotted gel was subjected to Northern analysis using labeled oligonucleotides specific for the different tRNAs. The cytosol-specific tRNA^Met-i^ was used to measure the extent of cytosolic contamination in the crude mitochondrial fractions. The tRNA^Ile^ on the other hand represents an efficiently imported tRNA and served as a positive control^27^.

**Figure 5:**
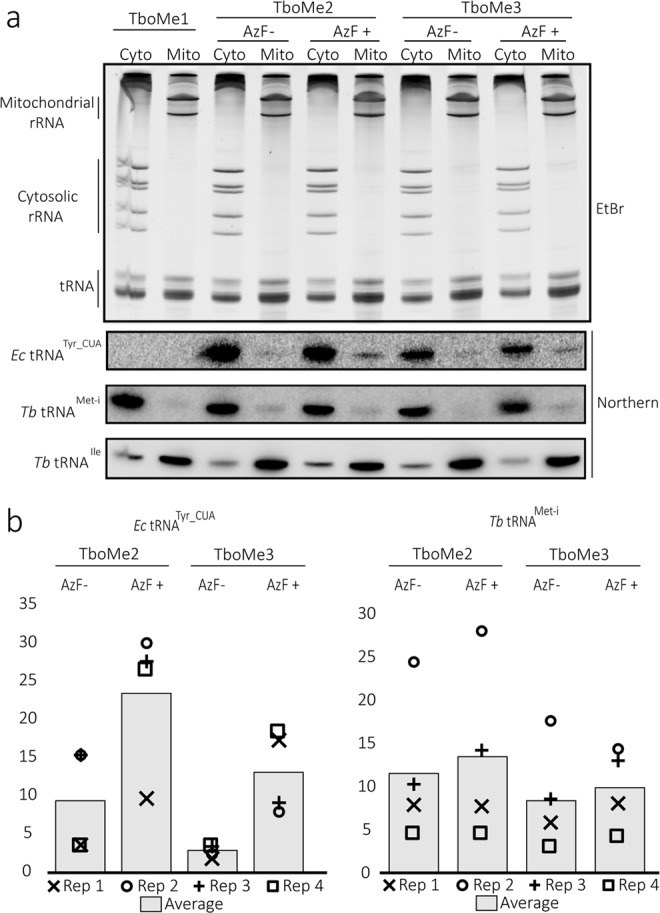
AzF-tRNA^Tyr_CUA^ is more effectively imported into the *T. brucei* mitochondrion than the uncharged form. (**a)** Top panel, cytosolic (Cyto) and mitochondrial (Mito) RNA fraction from the indicated cell lines (TboMe1, TboMe2, TboMe3) grown in the absence or presence of AzF were separated on a polyacrylamide gel containing 8 M urea and stained with ethidium bromide. The positions of the cytosolic and mitochondrial rRNAs as well as the tRNA region are indicated. Bottom panels, corresponding Northern blots of the gel shown in the top panel probed for tRNA^Tyr_CUA^, the cytosol-specific tRNA^Met-i^ and the efficiently imported tRNA^Ile^. **(b)** The mitochondrial signals of the Northern blots in bottom panels of (a) were quantified from four independent biological replicates (see Supplementary Figs S3–S5). The graph on the left depicts the mean (bar) as well as the individual values (symbols) for each of the four experiments of the absolute tRNA^Tyr_CUA^ signals present in the mitochondrial fractions of the indicated cell lines grown in the presence or absence of AzF. The graph on the right shows the corresponding absolute signals for tRNA^Met-i^, which serves as a control for cytosolic contamination.

A quantification of four independent experiments for each of the two cell lines, TboMe2 and TboMe3, shows that the mean of the absolute signal intensities, corresponding to the mitochondrial levels of tRNA^Tyr_CUA^, is 2.5 to 5 times higher in cultures that received AzF (Fig. 5b, left panel, see bars). The same is the case if each experiment is individually analyzed (Fig. 5b, left panel, see symbols). As a control for cytosolic contamination, the absolute signal intensities corresponding to mitochondrial levels of tRNA^Met-i^ were also determined. In contrast to tRNA^Tyr_CUA^ their levels remained essentially the same irrespective of whether the cells were grown in the presence or absence of AzF (Fig. 5b, right panel). The left and right panel in Fig. S1 shows the analogous quantifications for the cytosolic fractions, and the acid polyacrylamide gel in Fig. S2 documents the charging state of tRNA^Tyr_CUA^ in whole cell RNA extracted from the same cells used above.

In summary these results show that induction of aminoacylation by AzF induces import of a small fraction of the tRNA^Tyr_CUA^ into mitochondria, suggesting that aminoacylation of a tRNA is a prerequisite for it to be imported into mitochondria.

## Discussion

We have established a system allowing genetic insertion of a non-natural amino acid containing a photocrosslinking group at a predetermined position in a protein of choice in the procyclic forms of *T. brucei*. The system is based on the expression of an *E. coli* suppressor tRNA^Tyr_CUA^ and its corresponding *in vitro* evolved YRS, termed oMeYRS.

Northern analysis shows that the *E. coli* tRNA^Tyr_CUA^ is expressed. However, it is aminoacylated only when both oMeYRS is expressed and the non-natural amino acid is added to the culture. Finally, expression of full length nsGFP whose gene is interrupted by a UAG stop codon demonstrates that the aminoacylated *E. coli* tRNA^Tyr_CUA^ is functionally integrated into the trypanosomal translation system. Thus, the orthogonal *E. coli* tRNA^Tyr_CUA^/oMeYRS pair adds a new method to the molecular genetic toolbox of *T. brucei*. Since crosslinkers create covalent bonds between molecules, they can not only be used to probe for stable but also for transient interactions in protein complexes^28^. Recently insertion of photoactivatable crosslinkers into more than hundred different sites of the beta-barrel protein import pore Tom40 of yeast has been used to probe on a molecular level which path imported proteins take during membrane translocation^29^. The newly established orthogonal aminoacylation system could therefore in principle be used for similar studies in *T. brucei* which has highly unusual mitochondrial protein import machineries^7,10^. Moreover, ectopic expression of the gene containing the amber stop codon in its open reading frame could be combined with RNAi targeting of the 3′UTR of the endogenous mRNA. Upon induction with tetracycline the wildtype version of the protein would be essentially completely replaced by the one carrying the site-specific crosslinker.

The fact that the oMeYRS is expressed under the control of the tet-operator and repressor system^3^ allows the inducible increase of orthogonal aminoacylation. We have used this feature of the system to study the importance of aminoacylation for mitochondrial tRNA import. The results show that whereas the uncharged tRNA^Tyr_CUA^ remains in the cytosol, it gets imported into mitochondria after induction of aminoacylation. It has previously been shown that yeast and human tRNAs can be imported into mitochondria when expressed in *T. brucei*^30^. Our new results now show that this is also the case for a bacterial tRNA suggesting that any tRNA can be imported into mitochondria of *T. brucei*, irrespective of its evolutionary origin. Moreover, we provide the first direct evidence that aminoacylation is required for mitochondrial tRNA import. This is in line with previous studies which showed that binding to elongation factor 1a (EF1a) is a prerequisite for a tRNA to be imported into the mitochondrion of *T. brucei*^31^. EF1a is a cytosolic protein that is not imported into mitochondria. It therefore likely mediates the targeting step that directs tRNAs to mitochondria. Its involvement in this step explains why the initiator tRNA^Met^, which does not bind to EF1a, and the tRNA^Sec^, which has its own elongation factor, are not imported.

Because EF1a binding is selective for charged tRNA, the role of aminoacylation for mitochondrial import likely reflects the requirements of imported tRNAs to bind to EF1a. In that respect the situation is similar to the yeast system where a small fraction of a single tRNA^Lys^ isoacceptor is imported into mitochondria. Here charging is required for the tRNA^Lys^ to bind to the precursor of mitochondrial lysyl-tRNA synthetase with which it is translocated across the mitochondrial membranes using the protein import system^32–35^. However, while the protein import system is also required for mitochondrial tRNA import in *T. brucei*, protein import can be blocked without interfering with tRNA import indicating that the two systems use different mechanisms^15^.

Mitochondrial tRNA import is one, rather unconventional, example of how the orthogonal tRNA/aminoacylation system can be used to study trypanosomal biology. However, we are convinced that this new approach will be of great value to study many other unique features of trypanosomes.

## Materials and Methods

### Transgenic cell lines

The plasmid pAcBac2.tR4-OMeYRS/GFP* bearing *E. coli* YRS-6xHis Y37V, D182S, F183M and D265R (oMeYRS) as well as eGFP-cMyc-6xHis Y39TAG (nsGFP), in which the triplet coding for tyrosine at position 39 was replaced by TAG corresponding to a stop codon, was obtained from Addgene^19^. These genes were introduced into modified versions of the pLew100 plasmid for tetracycline-inducible expression in *T. brucei*, where the phleomycin resistance gene was replaced by either the blasticidine or puromycin resistance gene^3,36^. The oMeYRS gene was cloned into a pLew-100 derivative allowing N-terminal 3x-cMyc tagging that contained a puromycin resistance gene, while nsGFP was cloned without additional tagging into a pLew-100 derivative containing the phleomycin resistance gene.

A construct containing the *E. coli* tRNA^Tyr_CUA^ gene and 50 nt upstream as well as 30 nt downstream flanking sequence of *T. brucei* tRNA^Leu^ gene was synthesized commercially (Genescript). The 3′-acceptor CCA which is genetically encoded in the *E. coli* tRNA^Tyr_CUA^ gene was omitted. This construct was cloned into a pLew100-derived plasmid containing a blasticidine resistance gene where the splice acceptor site downstream of the twin tet operators was removed.

All three constructs were then sequentially transfected into procyclic *T. brucei* 29-13^3^ yielding the cell lines TboMe1 (expressing oMeYRS), TboMe2 (expressing oMeYRS and tRNA^Tyr_CUA^) and TboMe3 (expressing oMeYRS, tRNA^Tyr_CUA^ and nsGFP). TboMe0 (expressing the tRNA^Tyr_CUA^ only) was obtained by transfecting 29-13 with the tRNA^Tyr_CUA^ construct.

### Culture conditions

Cells were grown at 27 °C in SDM-79 liquid medium supplemented with 10% fetal calf serum. Cells were induced overnight by the addition of 2 µg/ml of tetracycline. O-methyltyrosine and AzF were added as indicated at 6.3 mM and 4 mM, respectively. When assaying the effect of aminoacylation on tRNA import, 2 mM AzF was used.

### RNA sample preparation

1.9 × 10^8^ cells were harvested and resuspended in 1 ml ice-cold phosphate-buffered saline. For the whole cell samples to assay for aminoacylation, 100 µl was removed and mixed with 400 µl guanidium isothiocyanate^37^ and then split into two tubes containing 200 µl each. When cytosolic and mitochondria-enriched RNA samples were required, the remainder of the cell suspension was extracted with digitonin as previously described^15^. Phenol-chloroform extraction and isopropanol precipitation were performed on the duplicate whole cell samples. The dried RNA pellets obtained were resuspended in 1.5 µl 20 mM sodium acetate pH 5.2 as well as in 1.5 µl 100 mM Tris-HCl pH 9 and heated at 50 °C for 20 min before adding 1.5 µl of formamide sample buffer to obtain the final untreated and deacylated whole cell RNA samples for loading on acidic urea-polyacrylamide gels.

### RNA separation and analysis

To separate aminoacylated from non-aminoacylated tRNAs, whole cell RNA samples were loaded onto 6.5% polyacrylamide gels (20 cm × 20 cm × 0.1 cm) containing 100 mM sodium acetate pH 5.2, 8 M urea and run at 200 V for approximately 6 hours at 6 °C in pre-chilled 100 mM sodium acetate pH 5.2 running buffer^23^. Gels were soaked in TAE buffer containing 40 mM Tris, 20 mM acetate, 1 mM EDTA and ethidium bromide for 10 min to visualize total RNA before wet transfer onto Genescreen Plus blotting membrane (Perkin-Elmer). TBE-buffered 8 M urea, 10% polyacrylamide gels were used to assay mitochondrial tRNA import as previously described^15^. Membranes were prehybridized and tRNAs hybridized with ^32^P-labelled DNA probes and then quantified as previously described^15^.

### Antibodies

Commercial antibodies were used in the following dilutions: mouse anti-GFP (Roche) 1:1000, mouse anti-EF1a (Merck) 1:10000, mouse anti-cMyc (Invitrogen) 1:10000 and goat anti-mouse IRDye 680LT (Licor) 1:20000. Electrophoresis in denaturing gels and Western blotting were performed as previously described^15^.

## Supplementary information

Supplementary Dataset 1
