# Supplementary material for: Inducible orthogonal aminoacylation demonstrates that charging is required for mitochondrial tRNA import in Trypanosoma brucei
Source: Sci Rep. 2019 Jul 25;9:10836. doi: 10.1038/s41598-019-47268-4 (PMC6658472; doi:10.1038/s41598-019-47268-4)
Supplement: Supplementary file 1 — Supplementary Dataset 1 [file 41598_2019_47268_MOESM1_ESM.docx]

**Inducible orthogonal aminoacylation demonstrates that charging is required for mitochondrial tRNA import in *Trypanosoma brucei***

J. L. Huot, S. Shikha and A. Schneider

Department of Chemistry and Biochemistry, University of Bern, Freiestrasse 3, Bern CH-3012, Switzerland;

Corresponding authors:

andre.schneider@dcb.unibe.ch, [jonathanlhuot@gmail.com](mailto:jonathanlhuot@gmail.com)

**Supplementary figures**

**
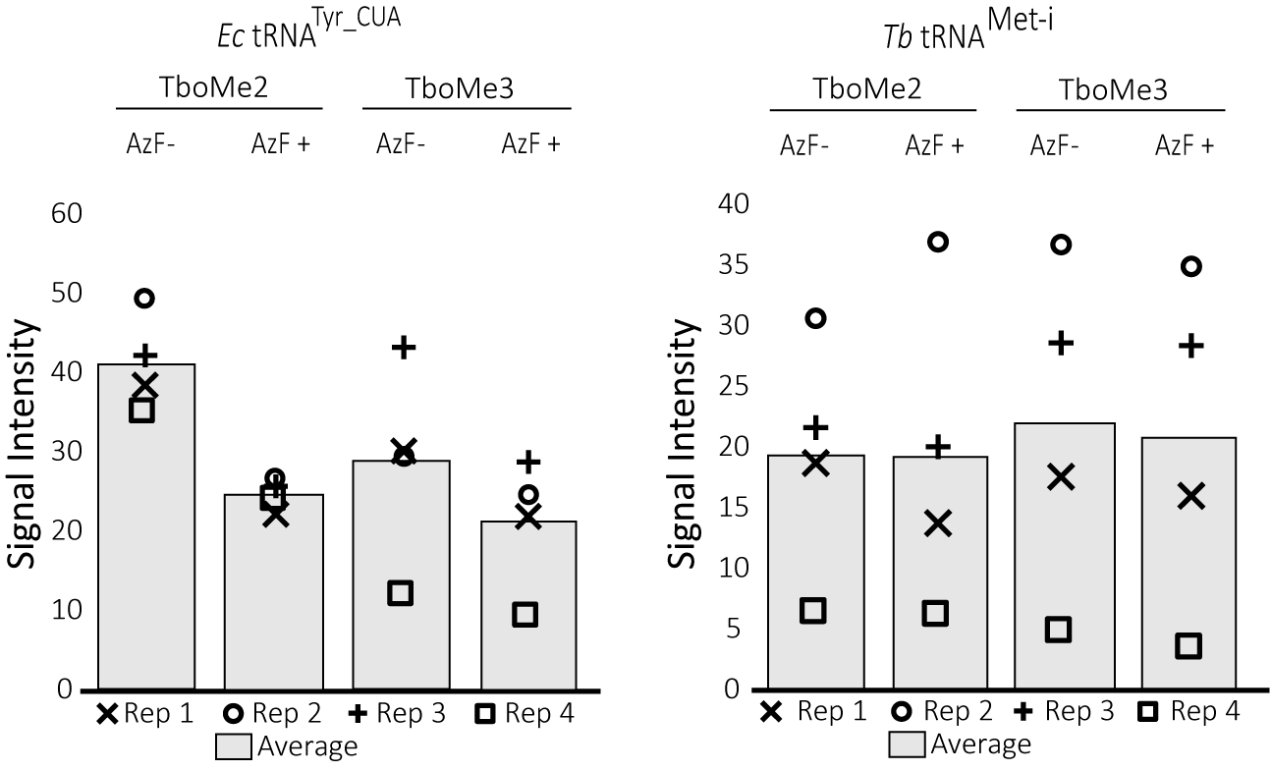
**

**Figure S1. The cytosolic signals of the Northern blots in bottom panels of Fig. 5A were quantified from four independent biological replicates (see supplementary Fig. S3-S5).** The graph on the left depicts the mean (bar) as well as the individual values (symbols) for each of the four experiments of the absolute tRNA^Tyr_CUA^ signals present in the cytosolic fractions of the indicated cell lines grown in the presence or absence of AzF. The graph on the right shows corresponding absolute signals for tRNA^Met-i^, which serves as a control for cytosolic contamination.

**
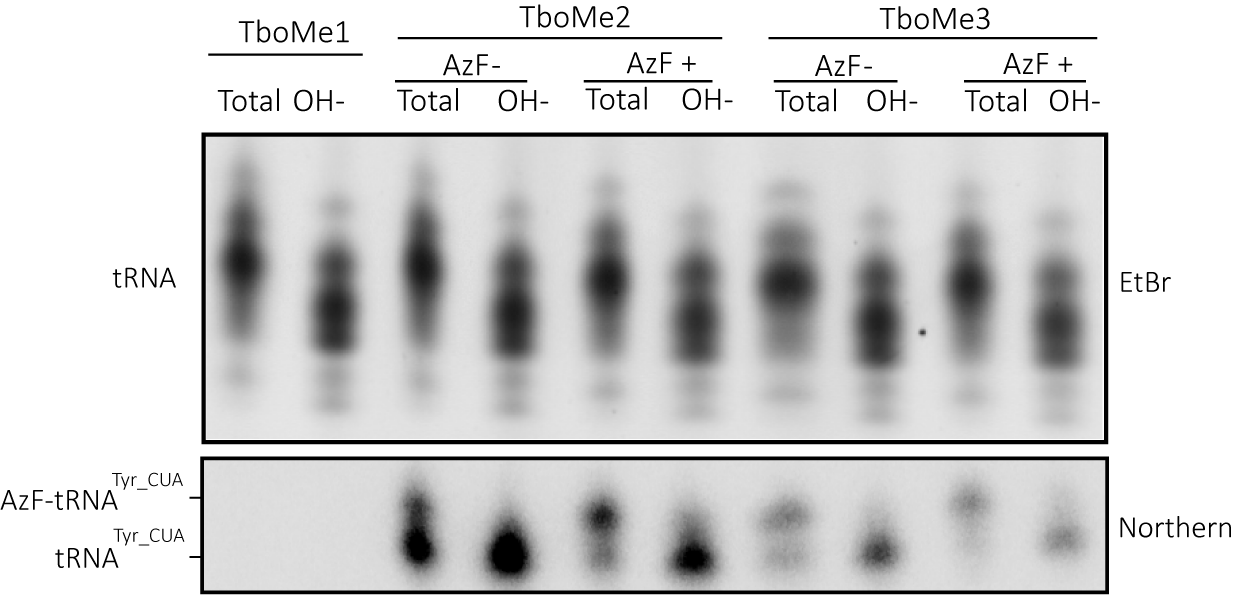
**

**Figure S2. Levels of charged tRNA^Tyr_CUA^ present in the total RNA fractions of the cell lines analyzed in Figure 5.** Acylated and deacylated (OH-) total RNA from the indicated induced (-/+ Tet) cells lines (TboMe1, TboMe2, TboMe3), grown with or without AzF, was separated on a long acidic polyacrylamide gel. Top panel, ethidium bromide stained gel. Only the region containing the tRNAs is shown. Bottom panel, corresponding Northern blot probed for tRNA^Tyr_CUA^.


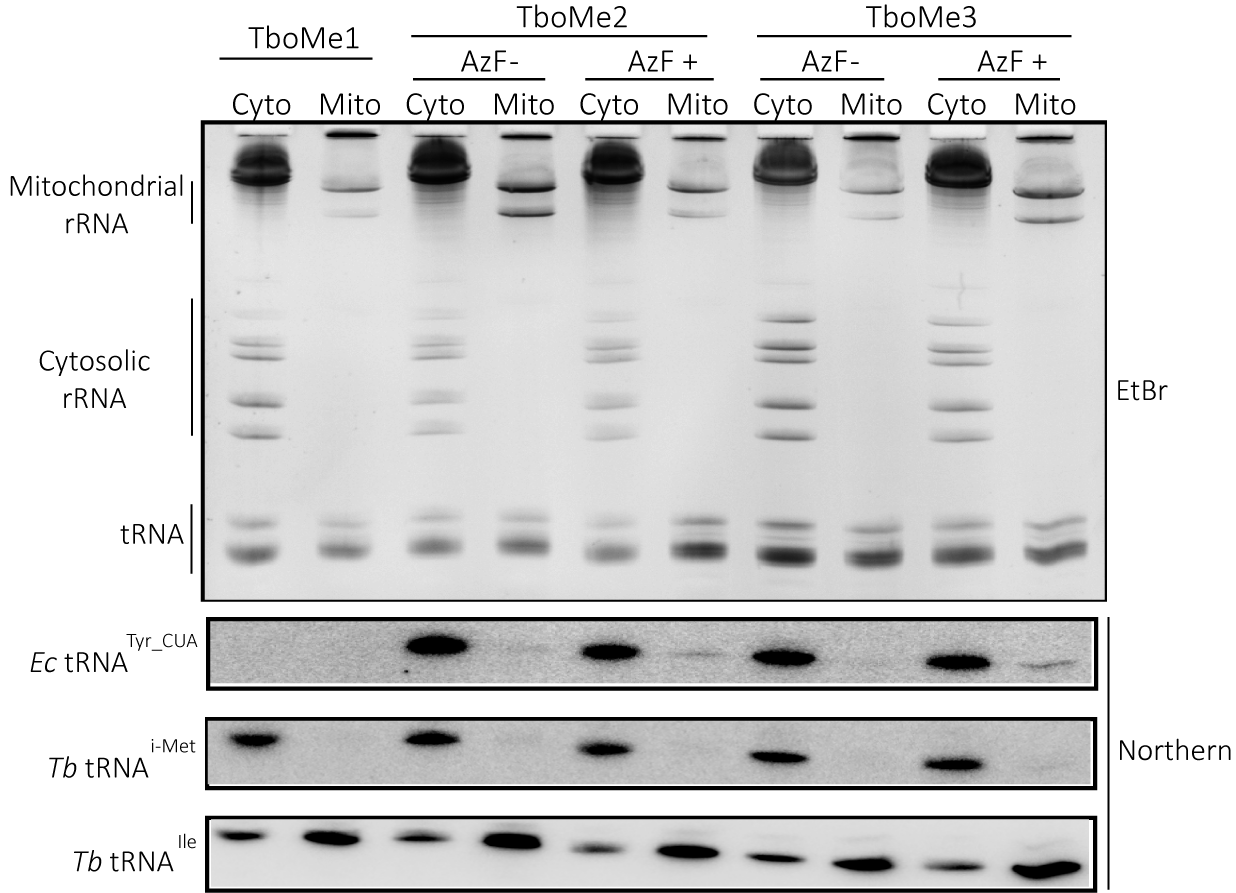


**Figure S3.** Replicate 2 of the data shown in Fig. 5a.


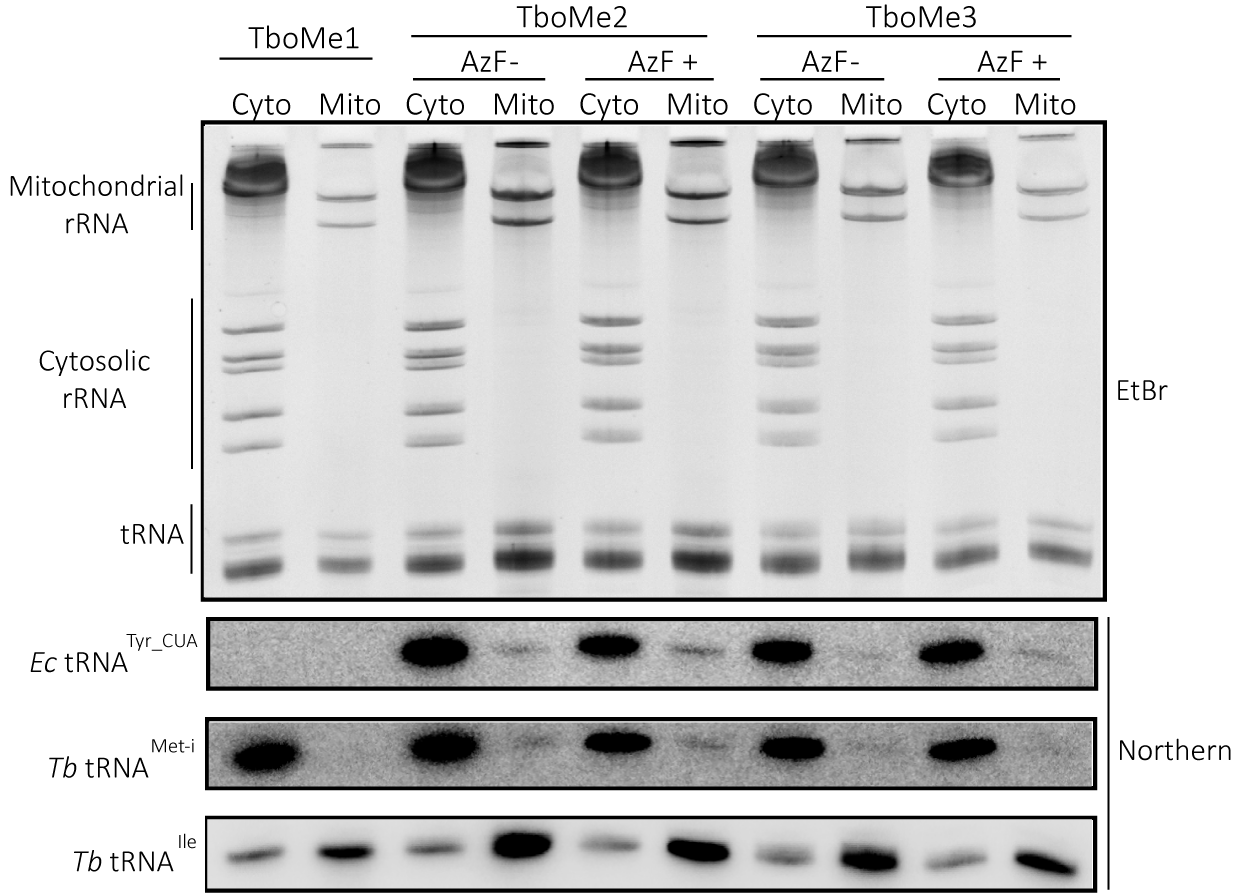


**Figure S4.** Replicate 3 of the data shown in Fig. 5a.

**
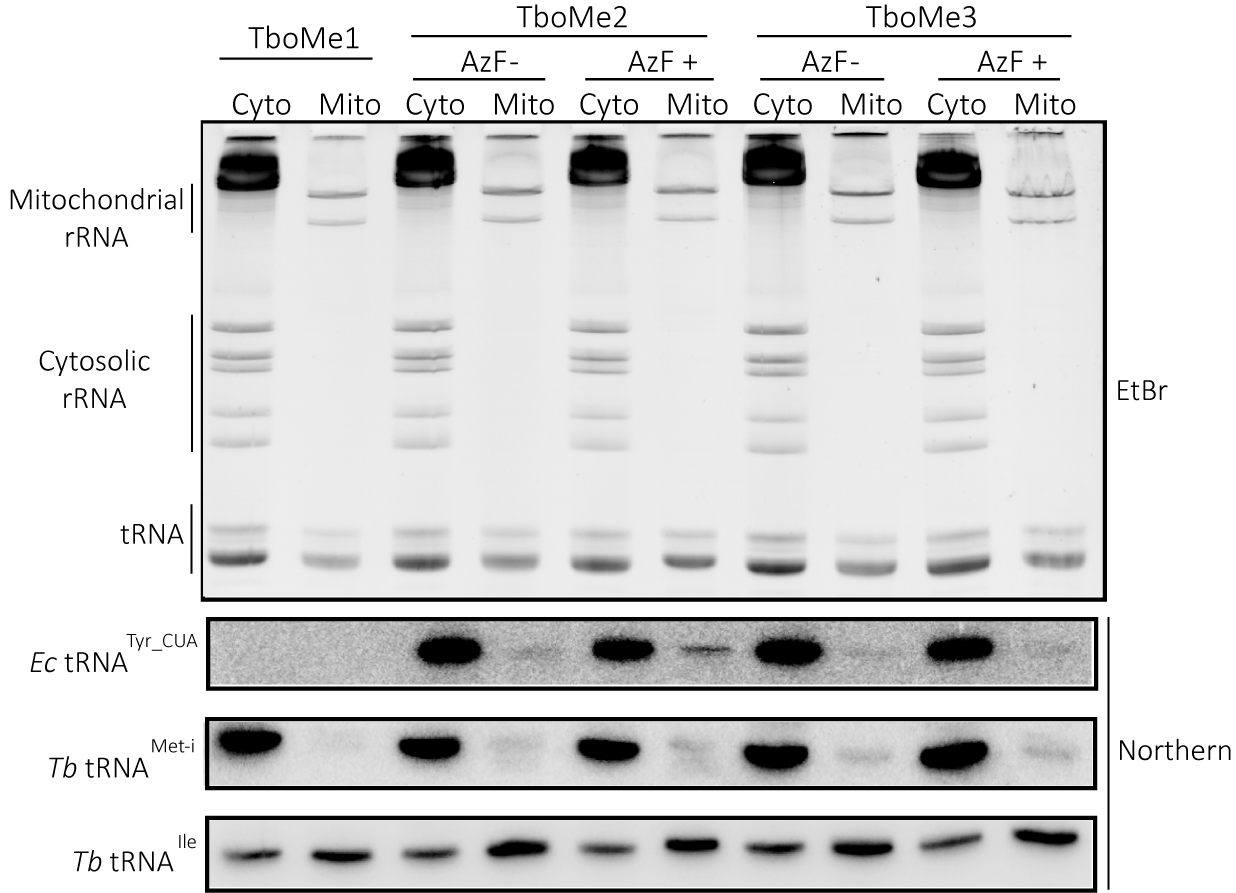
**

**Figure S5.** Replicate 4 of the data shown in Fig. 5a.

**Figure S6-S10. Full scans of all gels shown in the main figures of the manuscript**


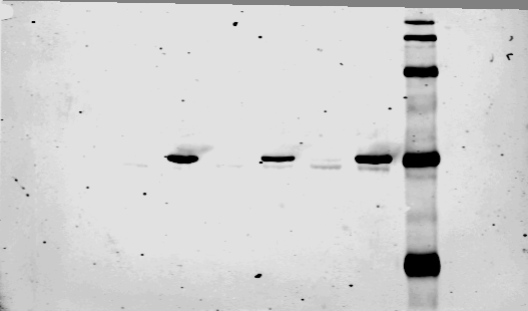


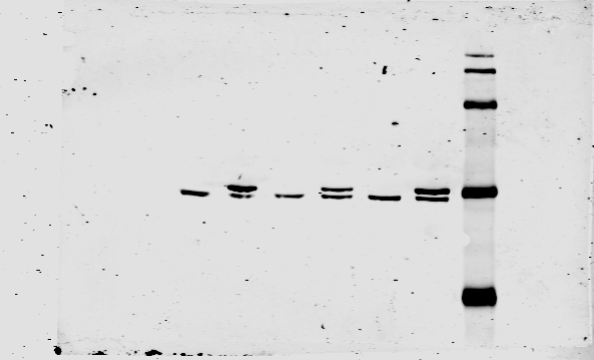


**Figure S6**. Full scans for Figure 2a. Anti-cMyc (top), anti-cMyc + anti-EF1a (bottom).





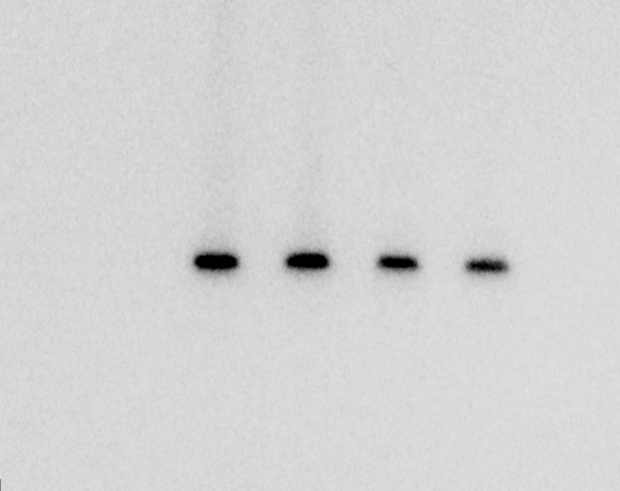


**Figure S7.** Full scans for Figure 2b. EtBr stain of RNA gel (top), northern blot probe against tRNA^Tyr_CUA^ (bottom).





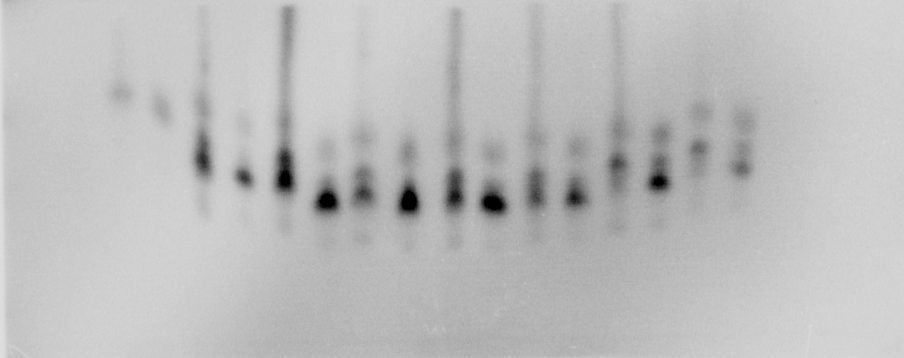


**Figure S8. Full scans for Figure 3. EtBr stain of RNA gel (top), and northern blot probe against** **tRNA^Tyr_CUA^ (bottom).** -Only the lower ~10cm of the gel (containing small RNAs) was transferred and blotted.


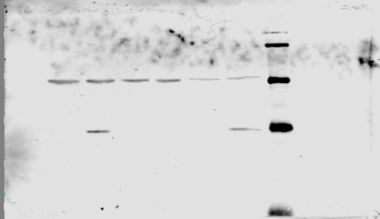


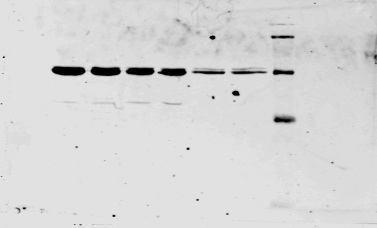


**Figure S9.** Full scans for Figure 4. Anti-GFP (top), anti-GFP + anti-EF1a (bottom).


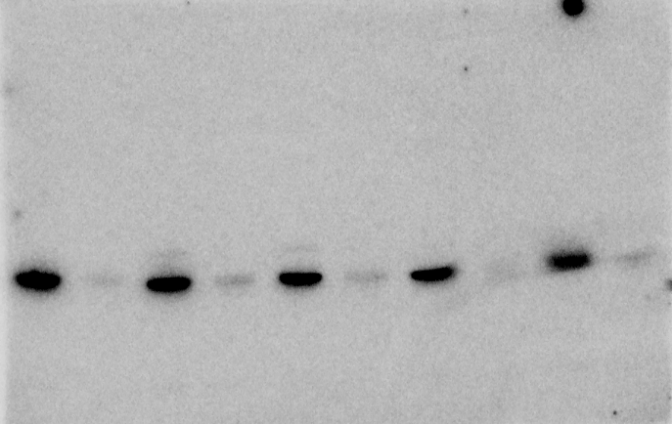

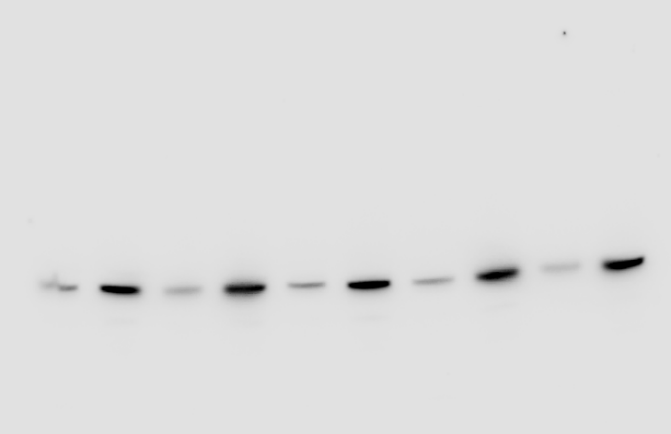

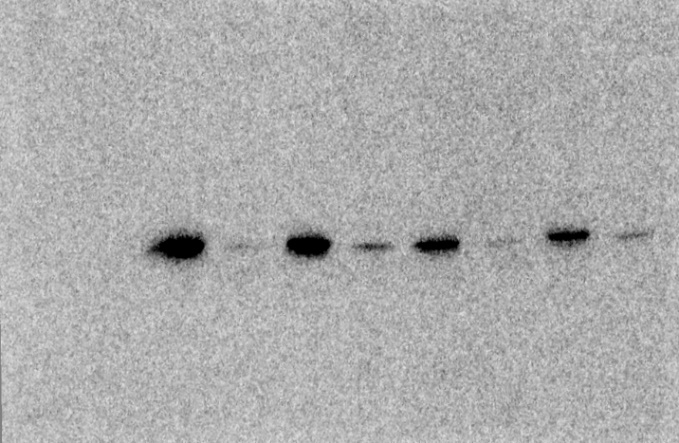




**Figure S10.** Full scans for Figure 5. Clockwise from top-left: EtBr-stained RNA gel, northern blot probe against tRNA^Tyr_CUA^, northern blot probe against Tb tRNA^Met-i^, and northern blot probe against Tb tRNA^Ile^.
